# Home-based exercise rehabilitation in addition to specialist heart failure nurse care: design, rationale and recruitment to the Birmingham Rehabilitation Uptake Maximisation study for patients with congestive heart failure (BRUM-CHF): a randomised controlled trial

**DOI:** 10.1186/1471-2261-7-9

**Published:** 2007-03-07

**Authors:** Kate Jolly, Rod S Tayor, Gregory YH Lip, Sheila M Greenfield, Michael K Davies, Russell C Davis, Jonathan W Mant, Sally J Singh, Jackie T Ingram, Jane Stubley, Andrew J Stevens

**Affiliations:** 1 Department of Public Health & Epidemiology, Public Health Building, University of Birmingham, Edgbaston, Birmingham, B15 2TT, UK; 2 Peninsula Medical School, University of Exeter, EX2 5DW, UK; 3 University Department of Medicine, City Hospital, Dudley Road, Birmingham, B18 7QH, UK; 4 Department of Primary Care & General Practice, University of Birmingham, Edgbaston, Birmingham, B15 2TT, UK; 5 Department of Cardiology, University Hospital Birminghan NHS Trust, Birmingham, B15 2TH, UK; 6 Sandwell and West Birmingham NHS Trust, Lyndon, West Bromwich, West Midlands, B71 4HJ, UK; 7 Dept Cardiac and Pulmonary Rehabilitation, University Hospitals of Leicester, UK

## Abstract

**Background:**

Exercise has been shown to be beneficial for selected patients with heart failure, but questions remain over its effectiveness, cost-effectiveness and uptake in a real world setting. This paper describes the design, rationale and recruitment for a randomised controlled trial that will explore the effectiveness and uptake of a predominantly home-based exercise rehabilitation programme, as well as its cost-effectiveness and patient acceptability.

**Methods/design:**

Randomised controlled trial comparing specialist heart failure nurse care plus a nurse-led predominantly home-based exercise intervention against specialist heart failure nurse care alone in a multiethnic city population, served by two NHS Trusts and one primary care setting, in the United Kingdom.

169 English speaking patients with stable heart failure, defined as systolic impairment (ejection fraction ≤ 40%). with one or more hospital admissions with clinical heart failure or New York Heart Association (NYHA) II/III within previous 24-months were recruited.

Main outcome measures at 1 year: Minnesota Living with Heart Failure Questionnaire, incremental shuttle walk test, death or admission with heart failure or myocardial infarction, health care utilisation and costs. Interviews with purposive samples of patients to gain qualitative information about acceptability and adherence to exercise, views about their treatment, self-management of their heart failure and reasons why some patients declined to participate.

The records of 1639 patients managed by specialist heart failure services were screened, of which 997 (61%) were ineligible, due to ejection fraction>40%, current NYHA IV, no admission or NYHA II or more within the previous 2 years, or serious co-morbidities preventing physical activity. 642 patients were contacted: 289 (45%) declined to participate, 183 (39%) had an exclusion criterion and 169 (26%) agreed to randomisation.

**Discussion:**

Due to safety considerations for home-exercise less than half of patients treated by specialist heart failure services were eligible for the study. Many patients had co-morbidities preventing exercise and others had concerns about undertaking an exercise programme.

## Background

Heart failure is a major cause of morbidity and mortality in westernised societies and has a poor prognosis [1-3] Patients with heart failure (HF) suffer from a variety of symptoms, many of which are non-specific and often result in a poor quality of life[4], with decreased exercise capacity being the main factor restricting daily activity.

Heart failure management in the UK is currently undergoing major changes as a result of evidence showing the benefits of specialist nurses in reducing hospital readmissions[5,6] Specialist heart failure nurses funded both by the British Heart Foundation and by local primary care trusts are becoming part of the accepted standard of care for patients with heart failure. Specialist heart failure nurses do not currently provide structured exercise training[6,7].

### Effectiveness of exercise rehabilitation

Four systematic reviews have addressed the effectiveness of exercise training for patients with heart failure [8-11]. A qualitative review in 2002 concluded that short-term exercise training in selected sub-groups of patients led to short-term improvements on quality of life and had some physiological benefits[8]. However, it was noted that most trials were of short duration, small scale and focussed on physiological changes rather than morbidity and mortality. They also had included a patient profile that is not representative of typical heart failure patients, being younger, predominantly male and without co-morbidities[8].

A Cochrane review which included 1126 patients in 29 randomised controlled trials (RCT) concluded that for patients with New York Heart Association (NYHA) class II or III heart failure, exercise training improved exercise capacity and quality of life in the short-term. Improvements in exercise capacity were greater in programmes of greater intensity and longer duration[10]. One study[11] using an end point of combined events and mortality found no difference in combined events (death or adverse events) in the exercise group (OR 0.98; 95% CI 0.61 to 1.32) compared to the control group. There was a non-significant trend towards a reduction in mortality (OR 0.71; 95% CI 0.37 to 1.02, p = 0.06)[11]. The ExTraMATCH collaboration undertook an individual patient data meta-analysis by combining the datasets from eight RCTs with a total of 801 patients[9]. This had access to longer-term follow-up data than was available to the previous meta-analyses and showed an overall reduction in mortality in the patients who received the exercise intervention (hazard ratio 0.65; 95% CI 0.42 to 0.92)[9].

Only one trial has compared exercise training in patients with a control group receiving a specialist heart failure nurse intervention[12]. This evaluated an eight-week hospital rehabilitation programme including exercise and education followed by a 16 week community supervised exercise programme in patients referred to a specialist heart failure nurse and reported significant improvements at 24 week follow-up in the Minnesota Living with Heart Failure Questionnaire (MLwHF) and EuroQol scores, the New York Heart Association (NYHA) class and distance walked on the 6-minute walk between the rehabilitation and specialist heart failure nurse care only[12]. The exercise intervention occurred at the same time as medication was optimised and there was a higher proportion of patients in the intervention arm on beta-blockade, which may have accounted for some of the difference. In addition the rehabilitation programme included an educational component and was not solely exercise-based.

### Mechanism of action of exercise

The majority of trials of exercise in patients with heart failure have reported physiological improvements, which are mainly due to peripheral adaptations [13-18] There is some evidence that exercise increases cardiac stroke volume[16,19-21] and reduces cardiomegaly[16]. Exercise training has been shown to lower resting heart rate and improve myocardial perfusion[22]. The mechanisms of these actions include an improvement in the neurohormonal abnormalities seen in heart failure, enhanced vagal tone, and changes in skeletal muscle type and function [13]. Favourable changes in the indices of heart rate variability have been reported, which indicates improved autonomic control of heart rate and has been associated with improved survival[23]. However, trials of exercise interventions rarely report data on the extent to which medication use changed, which would have affected the outcomes.

### Type of exercise and settings

Current recommendations for exercise training are based on experiences from a limited number of randomised controlled trials that have enrolled highly selected patients. Questions remain about the optimal training modality and intensity[24] but current evidence suggests that training should include aerobic and resistance components. Peripheral muscles should be trained without a significant increase in cardiovascular stress[25], using intermittent exercise and/or sequential training.

Whilst the National Service Framework for coronary heart disease suggests that cardiac rehabilitation should be considered for patients with heart failure[26], it is rarely provided in the UK. Indeed, there are no published data on the uptake and acceptability of exercise-based rehabilitation for heart failure, but poor uptake post myocardial infarction (MI) occurs particularly in women, the elderly and patients from minority ethnic groups [27-31]. and the profile of patients with heart failure is older and includes more women than is the case for MI. Only one entirely home-based exercise intervention trial (20 patients in home arm) has been reported, with significant improvements in quality of life compared to patients receiving usual care[32].

### Rationale for Birmingham Rehabilitation Uptake Maximisation study for patients with congestive heart failure (BRUM-CHF) study

Whilst there are a number of very small trials of exercise interventions for patients with heart failure, questions remain about the effectiveness of exercise interventions outside specialist research units and in a representative patient population[24]. The follow-up period needed to be of sufficient duration to measure hospital admissions and mortality. In addition, apart from one trial[12], the reported trials of exercise rehabilitation were all compared to 'usual care'. The latter provided in these control arms is inferior to the specialist heart failure nurse intervention now offered as part of modern heart failure management programmes. There is also no evidence as to whether the improvements in quality of life and mortality in the exercise intervention groups in the published trials, are a result of physiological improvements or from the close clinical monitoring received alongside the exercise. It may also be that lifestyle and drug management advice was imparted with the exercise intervention and these improvements would now result from the specialist nurse intervention. The cost-effectiveness of exercise interventions has been addressed by only one study[33], which had usual care as the control group. This concluded that a long-term hospital-based exercise programme for people with stable heart failure was a cost-effective intervention[33].

The BRUM-CHF study proposes to establish the additional benefits of exercise rehabilitation compared to current care that includes specialist heart failure nurses. Given the potential problems with the capacity of the hospital service and uptake in a more elderly and debilitated population than other patients receiving cardiac rehabilitation for MI or revascularisation, it will be important to have information about effectiveness, uptake and compliance in a predominantly home-based setting.

### Study aim

The primary research question seeks to evaluate whether there are additional benefits from exercise rehabilitation over specialist heart failure nurse management and to establish the cost-effectiveness and patient acceptability of a predominantly home-based programme of exercise rehabilitation. In addition, the study will investigate the patient experience of heart failure and rehabilitation whilst attaining information about effectiveness, uptake and compliance of patients in a predominantly home-based setting.

## Methods/Design

BRUM-CHF is a randomised controlled trial of exercise rehabilitation in heart failure comparing specialist heart failure nurse care alone with specialist heart failure nurse care plus a structured exercise intervention.

Prior to commencement of the study, ethical approval was obtained from Sandwell and West Birmingham Local Research Ethics Committee (Reference number: 03/10/708).

Randomisation was performed centrally by computer at the Birmingham Cancer Clinical Trials Unit, University of Birmingham. When a patient was identified as eligible for the study, and had given written, informed consent to take part, the research nurse telephoned the trials unit. The Trials Unit randomly allocated patients, using the method of minimisation, stratified by (i) NYHA group, (ii) presence or absence of atrial fibrillation and (iii) hospital site.

**Figure 1:**
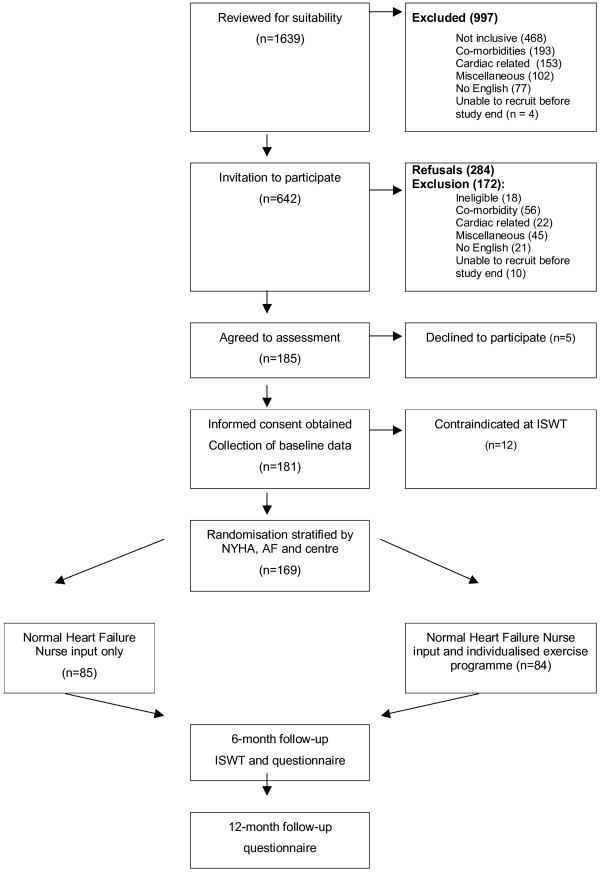
Identification and recruitment of participants to the BRUM-CHF study.

In one arm patients continued with "usual" specialist heart failure nurse care. In the other arm patients continued with "usual" specialist heart failure nurse care combined with the exercise programme. The trial design is summarised in Figure 1. Patients who refused randomisation continued with their normal specialist nurse care.

### Population

The two NHS hospital Trusts and one Primary Care Trust from which patients were recruited are in the West Midlands, UK, covering a combined multiethnic population of approximately one million with a high incidence of heart disease. Recruited patients had left ventricular systolic dysfunction with an ejection fraction of 40% or less measured quantitatively, or classed qualitatively as 'moderate to severe impairment' on an echocardiogram, and were identified from three specialist heart failure nurse services. Recruitment took place over 19 months. Inclusion and exclusion criteria are summarised in Table 1.

Details of all patients referred to the specialist heart failure services were collected and suitability for study participation identified. Potentially eligible patients were contacted, invited to participate and assessed prior to randomisation by an incremental shuttle walk test (ISWT) to determine their suitability for exercise and to exclude patients with symptomatic ischaemia, cardiac arrhythmias or marked hypotension on exercise[34,35].

Baseline data collected prior to randomisation included the NYHA class, left ventricular systolic ejection fraction, demographic details (age, sex, ethnicity), disease history (admissions for heart failure) and past medical history, current medication, and outcomes (see below).

### Interventions

#### Specialist heart failure nurse care

"Usual care" is that provided by primary and secondary care with specialist heart failure nurse input. To ensure homogeneity of the intervention received, especially in relation to heart failure nurse input and receipt of optimal medical treatment, patients were recruited from those referred to the specialist heart failure clinic. The heart failure nurses had all undergone training from the British Heart Foundation.

#### Exercise intervention

The exercise group received "usual care" as described above, plus an exercise programme which commenced with three supervised exercise sessions to provide the patient with confidence and to plan an individualised exercise programme. This was followed by a home-based programme with home visits at 4, 10 and 20 weeks, telephone support at 6, 15 and 24 weeks and a manual.

The home programme was predominantly aerobic training based largely on progressive walking with self-completion activity logs. Exercise prescription was based upon the participant's baseline exercise tolerance as measured by the incremental shuttle walking test. Performance on this test correlates well with peak VO_2 _[34,36] An individually prescribed walking programme was identified from this test at a speed that corresponded to 70% of peak performance. During the first two weeks of rehabilitation the speed of walking was secured by the patient with the help of the rehabilitation team. Targets were set weekly for the duration and frequency of walks, which largely depended on an individual's baseline exercise capacity. The programme aimed for the patients to achieve 20 to 30 minutes of walking 5 times a week after 10 weeks of rehabilitation. This was monitored with home training diaries.

Strength training was low intensity, using the patient's own body weight for resistance. The focus of the strength training was both upper and lower limb. The "milk bottle regime" currently used in the rehabilitation programme in Leicester are used. Patients complete sets of up to 10 repetitions of 8 key exercise using milk bottles filled with gradually increasing volumes (thus weights) of water. Targets are set and level of difficulty assessed. Individual exercises are omitted in patients with particular needs or difficulties.

Specific support for the exercise intervention ceases after six months but patients are encouraged to continue to maintain their activity levels.

### Outcome measures

Follow-up by postal questionnaire and clinical assessment occurs at 6 months and by postal questionnaire at 1 year.

The primary outcome measure is the Minnesota Living with Heart Failure Questionnaire (MLwHF) Questionnaire[37]. Secondary outcome measures at 6 months are (i) composite of death or admission with heart failure or myocardial infarction, (ii) admission with heart failure, (iii) mortality (all-cause and vascular), (iv) EuroQol[38], (v) HADS[39], (vi) blood pressure, (vii) self-reported physical activity, and (viii) distance walked on the ISWT[36]. At 12 months the ISWT and blood pressure measurements are omitted.

Process measures to determine the comparability of intervention received from the specialist heart failure service included self-reported smoking cessation, salt reduction and alcohol intake. Uptake and adherence to the exercise intervention are assessed by (i) attendance at the hospital training sessions, and (ii) patient completed exercise diaries at 4, 10 and 20 weeks in the intervention group.

The study could not be double blind because of the nature of the intervention. To reduce the potential for bias in measuring outcomes in the questionnaires we used validated measurement tools and aimed for clinical follow-up to be undertaken by an individual who had not provided the rehabilitation support and was blinded to treatment allocation. Determination of secondary end-points (hospitalisation for heart failure and vascular mortality) was by an independent committee blinded to treatment allocation.

### Sample Size

The overall power of the trial was determined on the basis of the primary outcome – health related quality of life.

To detect a 10 point difference between the 'usual care' and supervised exercise group on the MLwHFQ (13 points at 1 year in Belardinelli)[22] at the 5% significance level with 80% power, 140 evaluable subjects at 1 year were needed. Allowing for 20% mortality and loss to follow-up a total of 168 recruits (84 per group) were needed.

### Statistical analysis

All data will be analysed by intention to treat. Comparisons between the primary outcome measure will be made at two separate time points: 6 months and 1 year to assess both short and long-term effects of the exercise intervention.

For outcomes measured on a continuous scale (MLwHF questionnaire, HADS, Euroqol, blood pressure, self-reported physical activity, distance walked on ISWT), differences between the two groups will be investigated using a least squares regression framework. Differences in time related clinical outcomes (hospital readmission rates, mortality and composite of these) will be analysed using the Kaplan-Meier survival method and the two groups compared by the Log-Rank test. Multivariate regression methods will be used to take into account the baseline measurements for each patient. When baseline information is available this provides a more precise estimate of the treatment effect than either raw outcomes or change scores[40]. Results will be expressed as means and 95% confidence intervals.

### Qualitative study

The qualitative part of the study seeks to explore what patients with heart failure understand about their condition and what they think the role and potential outcomes of exercise are. The results of the qualitative study will complement and contextualise the quantitative findings e.g. people who do not take part may hold negative views about exercise or that it is dangerous, particularly in a home environment.

Interviews will be sought with purposive samples of (i) patients who declined to take part in the study, shortly after they have been invited; (ii) patients and their carers who were randomised to both exercise and control groups: during the exercise programme and at 6 months after recruitment.

We will use a maximum variety sample that represents the broadest range of social characteristics[41]. As the process of this type of research is iterative and reflexive it enables refinement of the course of enquiry in response to emerging findings i.e. new ideas mentioned by interviewed patients are integrated with current knowledge as the interviews progress and are included in subsequent interviews. This process is continued until interviewing is no longer generating new concepts and interviewing stops at this stage (i.e. theoretical saturation)[42]. To reach this stage it is generally estimated that between 20 and 30 interviews per group are necessary[43].

### Economic analysis

The costs of the alternative programmes will be assessed from two perspectives: NHS and societal. NHS costs will be based on resource inputs (time with the exercise rehabilitation nurse, travel time, drugs, use of NHS resources including any differences in hospital admissions linked to heart failure) costed up to include labour and overhead costs. National average unit costs will be used with exploration of local differences in sensitivity analysis. A cost-effectiveness analysis will be undertaken based on the outcome measures described above, with particular emphasis on deriving estimates of the incremental cost per QALY. Utility values will be based on the EQ-5D (or EuroQol) and life years from the mortality analysis described above.

Modelling will be used to extrapolate from the trial (both in time and by unit cost) and for sensitivity analyses. Resource use data will be collected as part of the overall data collection procedures. Bootstrapping will be used to explore differences in costs. Cost effectiveness results will be presented in the form of acceptability curves.

### Recruitment

The clinical notes of 1639 patients referred to the specialist heart failure nurse services were reviewed. Of these 997 (60.8%) were deemed not eligible, or had an exclusion criterion identifiable from the records (table 2). 468 (50.4%) had not been of NYHA class II or III or admitted with decompensated heart failure within the previous 24 months, or had an ejection fraction greater than 40%. A further 153 (15.3%) had a cardiac exclusion criterion, 204 (20.4%) a co-morbid condition precluding a home exercise programme.

The remaining 642 (39%) of patients were contacted by letter and invited to participate in the study once their heart failure was stable, and they were on maximised beta-blocker therapy. At this stage a further 172 (10.5% of total sample) were excluded and 284 (17.3%) declined to participate (figure 1).

The remaining 186 (12% of all initially reviewed patients) agreed to participate in the study, 2% of these failed to attend the assessment or changed their mind at assessment. The remaining 181 patients all gave written and informed consent and were assessed by incremental shuttle walk test for suitability before randomisation. We identified contraindication to home exercise in 12 of the assessed patients at the ISWT. Overall, only 10% of all currently alive patients referred to the three heart failure nurse services in the two years prior to commencing the study and during the 19 months of recruitment were actually randomised.

## Discussion

The BRUM-CHF study is novel in its evaluation of a predominantly home-based, structured exercise programme for patients with heart failure also receiving specialist heart failure nurse support. Recruitment to the trial has highlighted that such an exercise programme will be appropriate for a minority of patients with heart failure. The majority of patients seen by the specialist heart failure service were not eligible for the trial, due to factors that included insufficient or too severe disease severity or preclusion of safe home exercise. In addition, co-morbidities accounted for large numbers of patients not being suitable for exercise. The older age of patients with heart failure means that there will be a high level of co-morbidity, which was not an issue in previous trials of highly selected, atypical patients[8]. Poor recruitment to trials of patients with heart failure has been previously reported, with a high proportion declining to participate when contacted[44]. A trial of a nurse-intervention in primary care in the UK recruited 36% of those invited, compared with 26% of eligible patients in our study. Reasons for non-participation given included a perception of being too old, too unwell or too busy[44]. This is an issue which will need to be considered when planning rehabilitation services for patients with heart failure.

The qualitative study is being undertaken in a patient group which has received specialist heart failure nurse care. Previous research has highlighted the low understanding and knowledge about heart failure in patients with this condition[41,45-50], but this research was prior to the introduction of specialist heart failure teams. This research will provide information about whether the information needs of patients with heart failure are being met by the new specialist heart failure nurse services.

## Competing interests

The author(s) declare that they have no competing interests.

## Authors' contributions

KJ wrote the initial protocol and designed this study. RCD, GYHL, MKD, JS, AJS, JWM and SJS participated in the design of the study. RST wrote the statistical elements of the original protocol and contributed to the design of the study. SMG designed and wrote the qualitative element of the initial protocol. JTI and KJ wrote the initial draft of this paper. All authors read and approved the final manuscript.

## Pre-publication history

The pre-publication history for this paper can be accessed here:



## Figures and Tables

**Table 1:** Inclusion and exclusion criteria

**Inclusion Criteria**
Admitted with decompensated heart failure or had heart failure of severity NYHA II or III within the past 24 months
LVSF (left ventricular systolic function) with ejection fraction of <40% or moderate to severe impairment on 2D ECHO
Stable and compensated heart failure for at least 4 weeks
**Exclusion Criteria**
NYHA IV
Unable to speak English
MI or revascularisation within the past 4 months
Cardiac rehabilitation within the past 6 months
BP < 100 mmHg systolic, demonstrated postural hypotension, or fall in BP on exercise
Disabling CVA (stroke) within the past 6 months
Severe musculoskeletal problems preventing exercise
Unstable angina
Ventricular or symptomatic arrhythmias
Obstructive aortic valvular disease with a gradient of > 35 mmHg
Hypertrophic obstructive cardiomyopathy
Documented severe chronic obstructive pulmonary disease requiring medication
Case-note reported dementia or current severe psychiatric disorder

**Table 2:** Reasons for non-non-eligibility and exclusions from BRUM-CHF from case-note assessment in patients on specialist Heart Failure nurse case-loads

**Reason for exclusion/ineligibility**	**n**	**%**
**Not eligible**	468	46.9
**Co-morbidity**	204	20.4
Physically unable	124	
Cognitive dysfunction	38	
Airways disease	23	
Visual disability	11	
Malignancy	8	
**Cardiac related**	153	15.3
Recent stent	1	
Severe aortic stenosis/abdominal aortic aneurysm	10	
Unstable HF, arrhythmias, NYHA IV	81	
Implantable cardiac device	19	
Awaiting cardiac procedure: CABG/transplant/MVR	4	
Recent cardiac rehabilitation	1	
Recent myocardial infarction	1	
Recent cardiac transplant	1	
HOCM	1	
Postural hypotension	1	
Still being titrated on beta-blocker therapy	33	
**Miscellaneous**	91	9.1
Risk assessment – unsuitable for home-visits	21	
Unable to read or write	3	
Not living in area	46	
Transport difficulties with attending hospital	8	
Not contactable	9	
Other	4	
**Unable to speak English**	77	7.7
**Unable to recruit before study end**	4	0.4

**Total**	997	100
